# 

**Published:** 1850-02

**Authors:** 


### CONTENTS

OF THE

### MEDICAL EXAMINER.

NEW SERIES—NO. LXII.—FEBRUARY, 1850.

### ORIGINAL COMMUNICATIONS.

A case of Successful Operation for the Exsection of two Carious Ribs, and the lower portion of the Sternum. By the late George McClellan, M. D., of Philadelphia, - - - - - - 75

Philadelphia County Medical Society.—Discussion on the Powers of Quinine in Remittent Fevers, - - - - - - 78

Case of Chronic Farcy. Reported by John H. Weir, M. D., Philadelphia, - - - - - - - - - - - - 96

Treatment of Scarlatina. By G. W. Brown, of Port Carbon, Pennsylvania, - - - - - - - - - - - - 98

Ulceration of the Cœcum,—Hæmorrhage,—Death. By H. A. Ramsay, M. D., Raysville, Georgia, - - - - - - - - 100

### BIBLIOGRAPHICAL NOTICES.

Inflammation: its Symptoms, Causes, and Treatment philosophically considered. By J. P. Batchelder, M. D., - - - - 102

Catalogue of Skulls of Man and the Inferior Animals, in the collection of Samuel George Morton, M. D., Penn. and Edinb., President of the Academy of Natural Sciences of Philadelphia, &c. &c., 114

### EDITORIAL.

National Convention for the Revision of the Pharmacopœia, - - 118

Delegates to the Convention for Revising tde United States Pharmacopœia, - - - - - - - - - - - 118

Delegates to the American Medical Association, - - - - 118

" State Society, - - - - - - - - 118

Death of Dr. Samuel B. Woodward, - - - - - - 119

" John F. Brooke, U. S. N. - - - - - - - 119

" L. M. Kane, - - - - - - - - - 119

Insane Asylums, - - - - - - - - - - - 119

Medical Society of the State of Pennsylvania, - - - - 120

### RECORD OF MEDICAL SCIENCE.

### ANATOMY AND PHYSIOLOGY.

Lectures on the processes of Repair and Reproduction after Injuries. Delivered at the Royal College of Surgeons of England. By James Paget, F. R. C. S., Professor of Anatomy and Surgery to the College, - - - - - - - - - - 121

### PATHOLOGY AND PRACTICE OF MEDICINE.

On Nervous or Convulsive Cough. By M. Sandras, - - - 127

M. Edouard Vandezande on Colchicum in Dropsy, - - - 128

#### MATERIA MEDICA AND THERAPEUTICS.

Experimental Researches on the action of Quinine, especially in large doses. A Memoir submitted to the Academy of Science. By M. Brecquet. Report of MM. Andral, Rayer, and Lallemand, 131
Remedy for Hydrophobia. By Rocher d'Héricourt, - - - 132

#### SURGERY.

On the use of Nitrate of Silver in Lacerated Wounds. By John Higginbottom, Esq., F. R. C. S., Nottingham, - - - - 133
Prevention of the entrance of Air when removing Fluid from the Pleuræ, Peritoneum, and Cavities of Abscesses, - - - 135

#### OBSTETRICS.

Case of Placenta Prævia. By W. F. Vidal, M. R. C. S., Aveley, Essex, late House Surgeon to the London Hospital, - - 135

